# Dietary ARA Improves COX Activity in Broodstock and Offspring Survival Fitness of a Model Organism (Medaka *Oryzias latipes*)

**DOI:** 10.3390/ani10112174

**Published:** 2020-11-21

**Authors:** Agata Kowalska, Maciej Kamaszewski, Marta Czarnowska-Kujawska, Piotr Podlasz, Radosław K. Kowalski

**Affiliations:** 1 Department of Fisheries Bioeconomics, Inland Fisheries Institute, 10-719 Olsztyn, Poland; a.kowalska@infish.com.pl; 2 Department of Ichtiology and Biotechnology in Aquaculture, Warsaw University of Life Sciences, 02-786 Warsaw, Poland; maciej_kamaszewski@sggw.edu.pl; 3 Department of Food Science, University of Warmia and Mazury, 10-718 Olsztyn, Poland; marta.czarnowska@uwm.edu.pl; 4 Department of Pathophysiology, Forensic and Administration of Veterinary Medicine, University of Warmia and Mazury, 10-714 Olsztyn, Poland; piotr.podlasz@uwm.edu.pl; 5 Department of Gamete and Embryo Biology, Institute of Animal Reproduction and Food Research of Polish Academy of Sciences, 10-748 Olsztyn, Poland

**Keywords:** *Oryzias latipes*, arachidonic acid, cyclooxygenases, reproduction parameters, larval quality

## Abstract

**Simple Summary:**

The impact of arachidonic acid in broodstock diets on cyclooxygenase activity and quality of offspring was investigated on model fish species. The results have shown that arachidonic acid in the broodstock diet improves sperm motility parameters. The cyclooxygenase activity in the liver of females increased with the amounts of arachidonic acid in diets. The arachidonic acid bioconversion activity during the reproductive season via cyclooxygenase activity is involved in female fertility and embryo and larval survival and growth.

**Abstract:**

A 3-week feeding trial was conducted in medaka broodstock (age five months) to examine the effect of dietary arachidonic acid (ARA) level (range: 4–23 mg g^−1^ of total fatty acids (TFAs)) on fertility, cyclooxygenase (COX) activity, egg size, sperm motility parameters, hatching rate and weight of hatch, survival and growth of larvae. After spawn induction and dietary exposure to 4 mg ARA g^−1^ TFA, broodstock were fed a diet containing ARA in the amounts: 4 (continued, as control), 5, 14 and 23 mg g^−1^ TFA. COX1 activity in the liver and the number of COX2-positive cells in the ovaries was increased in females fed the diets containing the two highest amounts of ARA. The highest sperm motility parameters were observed in males fed a diet containing 23 mg ARA g^−1^ TFA. The hatchability rate and bodyweight of hatchlings were higher in the group obtained from broodstock fed a diet containing 23 mg ARA g^−1^ TFA (79% and 0.66 mg fish^−1^, respectively) compared with 4 mg ARA g^−1^ TFA (50% and 0.40 mg fish^−1^). The average mortality of offspring obtained from this group at 7 days post hatching was significantly higher than that of all other groups.

## 1. Introduction

Various fat sources are currently used in fish feed. The replacement of fish oil by vegetable oils changes the nutritional value in terms of the LC-PUFA (long chain poly-unsaturated fatty acids) amount [1] and may be linked to a deficit of essential fatty acids (EFAs) and the balance of eicosapentaenoic acid (20:5 n-3, EPA), docosahexaenoic acid (22:6 n-3, DHA) and arachidonic acid (20:4 n-6, ARA) in the fish body and gametes [2,3,4,5,6]. Dietary supplementation of components with EFAs that are crucial to reproduction is justified and can lead to improvements in reproductive results.

The ARA content of the broodstock diet may be a limiting factor for successful reproduction. It is known that ARA is a precursor for the synthesis of eicosanoids of the 2-series prostaglandins and can therefore stimulate testicular testosterone, the production of steroid hormones, gonad development, gamete maturation, ovulation and the spermatogenic process [7,8,9,10]. ARA is involved in modulation of the immune system, thereby increasing overall resistance and, consequently, offspring survival [11]. Therefore, ARA supplementation of broodstock diet can provide better possible conditions for spawning and larval culture.

Low content of ARA in broodstock diets has an impact on the long-chain polyunsaturated fatty acids (LC-PUFAs) ratio. This can cause reproductive disorders by inhibiting the synthesis of 2-series prostaglandins [12]. The synthesis of this eicosanoid is carried out through conversion of ARA by cyclooxygenase enzymes (COX1 and COX2). The roles of COX1 and -2 in the fish reproductive system remain unclear [13] and their activities are variable [14]. Thus, it was considered purposeful to evaluate the effect of ARA addition to broodstock diet on hepatic activity and localization in the ovary of cyclooxygenases (COX1 and COX2), female fecundity, egg size, sperm motility, embryo survival, hatching rate, survival and growth of larvae in medaka.

Medaka (*Oryzias latipes*) is a valuable model fish species in aquaculture for the study of reproduction [15,16]. Medaka is an attractive model organism for the evaluation of the spawning cycle as well as the parental and progeny generations because of its easily induced spawning stage and short maturation time. The rearing techniques used for broodstock and larvae, obtaining oocytes, embryo handling and observation improve the analysis of reproductive parameters [15]. Consequently, this model species has been chosen to investigate the effect of dietary administration of ARA in broodstock diet supplemented with pure (≥99%) ARA on fish reproduction.

## 2. Materials and Methods

Procedures were carried out in accordance with the Local Committee on the Ethics of Animal Experiments in Olsztyn, Poland No 10/2009/N.

### 2.1. Broodstock

The experimental material comprised medaka originally obtained from fish derived from the National Institute of Natural Science (Tokyo, Japan). Ongrowing medaka were reared according to the requirements set out experimentally for this species [15]. The fish were fed commercial feed recommended for medaka (TetraMin, Melle, Germany), which contained 4 mg ARA g^−1^ of total fatty acids (TFAs). After the medaka had reached maturity (age 5 months post-hatch), an average body weight (BW) of 0.45 g and body length (TL) of 3.5 cm, the fish were stocked into 12 tanks (20 L glass aquaria), each equipped with a filter and impeller pump (Aqua Szut, Wrocław, Poland), heater (Aquael, Warsaw, Poland) and external light (Palm Light, AZOO, San Diego, CA, USA). The ratio of females to males was 1:1 (20 individuals per aquarium). A photoperiod of LD 14:10 was applied to initiate spawning. Light intensity measured at the surface of the rearing aquarium was 650 lux. The water temperature, oxygen content and pH were as follows: 27.0 ± 0.1 °C, 5.7 ± 0.4 mg O_2_ L^−1^ and pH 6.2–6.8. The animals were maintained in a balanced salt solution (BSS) 0.65% NaCl, 0.04% KCl, 0.02% MgSO_4_ 7H_2_O, 0.02% CaCl_2_ 2H_2_O [15]. Fish were reared for 30 days and fed twice daily (at 10.00 a.m. and 3.00 p.m.) a total amount of 37 mg g^−1^ BW. No broodstock mortality was observed in any of the dietary treatments during the experiment.

### 2.2. Experimental Diet

To prepare experimental diets, a commercial feed recommended for medaka (TetraMin, Melle, Germany) containing 4 mg ARA g^−1^ TFA was used and supplemented with different amounts of ARA (≥99%; Sigma-Aldrich, St. Louis, MO, USA) (Table 1). The fish were divided into four treatment groups, each dietary treatment was carried out in triplicate (*n* = 3). At the beginning (9 days), all fish in 12 glass aquaria were fed a commercial diet (TetraMin, Germany) containing 4 mg ARA g^−1^ TFA. During next 21 days of the experiment, the feeding of fish was continued with commercial feed containing 4 mg ARA g^−1^ of TFA (control group) or various ARA level as follows: 5, 14 and 23 mg g^−1^ of TFA. To incorporate ARA into the diet, the ARA was initially diluted with ethanol (96%). Appropriate aliquots of ARA were added to the feed and mixed well. To achieve different ARA concentrations in the diet, the ARA aliquots were additionally diluted before addition. The amount of aliquots added to the diet was the same in all groups. After ARA addition, feed was dried at room temperature for 2 h. The diets were analyzed to determine the contents of particular fatty acids (mg g^−1^ of total fatty acid (TFA)). Qualitative and quantitative analyses of fatty acids were conducted on cold-extracted lipids according to methods described in Folch et al. [17]. The fatty acids were methylated using a mixture of chloroform, anhydrous methanol and sulfuric acid (100:100:1) [18]. Chromatographic separation was performed with an Agilent Technologies 6890N gas chromatograph with a flame ionization detector (FID) (Agilent Technologies Inc., Santa Clara, CA, USA). The signal was registered by a Philips recorder with 1 mV full-scale sensitivity at a tape speed of 10 mm min^−1^. Individual acids were identified by comparing retention times with standards from Supelco (Bellefonte, PA, USA).

### 2.3. Evaluation of the Reproductive Effect

On the last day of broodstock rearing, fish were anaesthetized on ice for 1 min. Males were sacrificed and their testes were dissected for the analysis of sperm quality. Females (BW ± 0.01 g) and their livers (LW ± 0.001 g) were weighed to determine the values of the hepatosomatic index (HSI = LW × 100/BW; %). After weighing, livers were collected for the analysis of COX activity.

#### 2.3.1. Sperm

After dissection, the testes were removed and transferred individually to 1.5 mL tubes containing 10 μL of Hanks’ balanced salt solution (HBSS; 0.137 M NaCl, 5.4 mM KCl, 1.3 mM CaCl_2_, 1.0 mM MgSO_4_, 0.25 mM Na_2_HPO_4_, 0.44 mM KH_2_PO_4_, 4.2 mM NaHCO_3_, and 5.55 mM glucose, pH = 7.2) supplemented with 50 mM trehalose and 0.5% of BSA as an extender [16]. In this buffer, sperm remains motile about 2 days from the collection (unpublished data). Sperm motility was observed 1 h after sample collection. Activated sperm were placed on Teflon-coated 12-well glass slides (Tekdon, Inc., Myakka City, FL, USA) and covered with standard coverslips. The mixture of sperm and Hanks’ balanced salt solution (1 μL) was placed on a slide and sperm movement was recorded six seconds after activation. Video recordings for computer assay sperm analysis (CASA) were made using a microscope with a 20× negative phase. Recordings were made with a Basler a202K digital camera (Basler AG, Ahrensburg, Germany) integrated with an Olympus BX51 microscope (Olympus, Tokyo, Japan). The recording speed was 47 frames per second. The first 200 frames from each recording were analyzed using the program Image House CRISMAS Company Ltd., Copenhagen, Denmark The percentage of motile sperm (MOT, %), progressive motility (%), average path velocity (VAP, μm s^−1^), straight line velocity (VSL, μm s^−1^), curvilinear velocity (VCL, μm s^−1^) and linearity (LIN = 100 × VSL/VCL, %) were analyzed to compare sperm quality among experimental groups.

#### 2.3.2. COX Activity

For the removal of remnants of the surrounding tissue and blood, livers were washed in Tris-HCl buffer (100 mM, pH 7.4) and homogenized in buffer (100 mM Tris-HCl, 1 mM EDTA, pH 7.8) in a volume of 5 mL g^−1^ of the liver, and centrifuged at 10,000 *g* for 15 min at 4 °C. The supernatant was frozen at −80 °C for analysis.

COX activity was determined using the putty COX Activity Assay Kit (Cayman Chemical, Ann Arbor, MI, USA) and measured by determining the transformation of arachidonic acid to PGG2 followed by the quantitative conversion of PGG2 to PGH2 using the colorimetric substrate TMPD. To determine the activities of individual COX enzymes, COX1- and COX2-specific inhibitors, SC-560 and DuP-697, respectively, were used. The change in color intensity determined the amount of TMPD substrate consumed in the reaction. After a 5 min incubation at 25 °C, samples were measured by colorimetry at a wavelength of 590 nm. COX1 and COX2 activities were determined by subtracting the activity obtained in the presence of inhibitors from the total activity (in samples without inhibitor). All tests were analyzed in duplicate, and the mean of the two readings based on the model were used to obtain the activity of COX enzymes.

#### 2.3.3. Immunohistochemistry Detection of COX2

At the end of the experiment, 15 female medaka from each experimental group (five individuals from each aquarium) were anaesthetized and fixed in Bouin’s solution and used for immunohistochemical purposes. Fixed samples were dehydrated in a graded series of ethanol, embedded in Paraplast and sectioned into 5 μm transverse sections using a microtome (Leica RM 2265, Leica Microsystems, Nussloch, Germany).

To detect COX2 in the medaka ovary, histological slides were deparaffinized in xylene and rehydrated using a gradient of ethanol. Endogenous peroxidase was blocked with 3% hydrogen peroxide. The histological slides were rinsed in Tris buffer (pH 8.0) (T-6664, Sigma). Slides were blocked with horse serum and incubated with primary antibody overnight at 4 °C. The polyclonal anti-mouse COX2 (Cayman Chemical, Ann Arbor, MI, USA) antibody was applied at a 1:200 dilution. The visualization process was completed according to the manufacturer’s instructions using a DAKO EnVision System-HRP (DAKO, Glostrup, Denmark). The cell nucleus was stained using Harris hematoxylin. A negative control (without antibody) was included in immunohistochemical analysis. Slides were dehydrated in a series of ethanol, rinsed in xylene and mounted with DPX (Sigma).

Microscopic observations were carried out using a Nikon Eclipse 90i microscope and Nikon Digital Sight DS-U1 camera (Nikon Corporation, Tokyo, Japan). Photographs were taken using the program NIS-Elements AR 2.10 (Nikon Corporation, Tokyo, Japan). Iwamatsu et al. [19] categorized the oocyte development of medaka into 10 stages. The COX2-positive cells were counted in the germinal epithelium and calculated to the 1000 µm^2^ area of this epithelium (10 areas from each analyzed fish). Around the large follicles, COX2-positive cells were counted in the granulosa layer and calculated to 100 µm of granulosa cells and theca cells surrounding follicle in vitellogenic and postvitellogenic phase (10 areas from each analyzed fish).

#### 2.3.4. Eggs

Eggs were collected from the abdomens of females 2 h after the start of the light period and fecundity (number of eggs female^−1^) was calculated. The eggs were placed on plastic 90 mm diameter Petri dishes with the number of eggs not exceeding 60 eggs plate^−1^ (i.e., clusters from two to three females) and incubated in embryo culture medium (25 mL plate^−1^) with methylene blue to prevent fungus (0.5 ppm) at 23.3 ± 0.25 °C [15]. Embryo survival was defined at the eyed-egg stage (using a stereoscopic microscope). During experimental diet feeding of broodstock, at day 7, reverse-fertilized eggs were collected from nine females from each experimental group (three females from each aquarium) and measured (egg diameter ± 0.01 mm) using the computer program Nis-Elements (Nikon, Tokyo, Japan).

### 2.4. Larvae

After hatching larvae were reared in 12 glass aquaria (30 cm × 40 cm × 30 cm, the volume of the reservoir: 20 L; 100 individuals in each aquarium). The larvae were fed twice per day (10:00 a.m. and 3:00 p.m.), with only artificial feed recommended for medaka larvae (TetraMin, Germany) ad libitum (0.3 g aquarium^−1^ per day). The amount of ARA in the larval diet was determined by Folch et al. [17] and Peisker [18] and was 4 mg ARA g^−1^ TFA. Uneaten feed was removed every day and one-fifth of the water (4 L in each aquarium) was replaced with fresh water daily. The physicochemical parameters of the water were monitored twice a week. The temperature and pH of the aquaria ranged from 27.0 ± 0.2 °C to 27.2 ± 0.4 °C and from pH 6.52 ± 0.12 to 6.68 ± 0.18, respectively.

Larval growth and mortality were monitored for three weeks post-hatch. Initial weight (BW ± 0.1 mg) was determined by measuring the cumulative mass of hatch samples taken from each dietary treatment (in triplicate, three samples and 10 specimens for each sample). Final weight (after three weeks of rearing) was determined by individual fish weight (60 fish from each treatment, 20 individuals per aquarium). Individual length of larvae (TL ± 0.01 mm) was measured with using a Nikon E600 (Tokyo, Japan) light microscope and the NIS-Elements F2.30 v. 2.21 (Nikon, Tokyo, Japan) program. Dead fish were removed every day and mortality was recorded after one, two and three weeks.

### 2.5. Statistical Analysis

The results of all measurements and calculations were subjected to statistical analysis using GraphPad Prism program software (Soft. Inc., Avenida de la Playa la Jolla, CA, USA). Means were compared by single-factor analysis of variance (ANOVA). When statistically significant differences were confirmed among dietary treatments (*p* ≤ 0.05), Tukey’s post hoc test was applied. All values expressed as percentages were transformed with arcsin before statistical processing.

## 3. Results

### 3.1. Effect of the ARA Diets on Broodstock and Gametes

**Figure 1:**
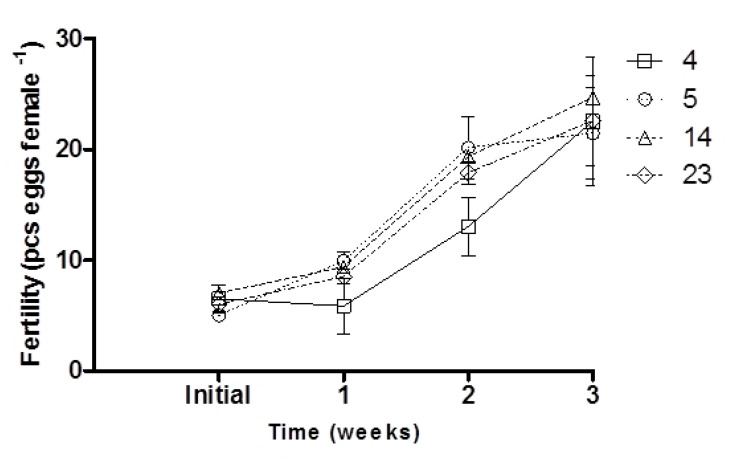
Effect of dietary ARA level (4, 5, 14, 23 mg ARA g^−1^ TFA) in broodstock diet on the fertility of medaka females during three weeks of experimental diet feeding. Values are means ± standard deviation (SD); each dietary treatment was carried out in triplicate (*n* = 3).

**Figure 2:**
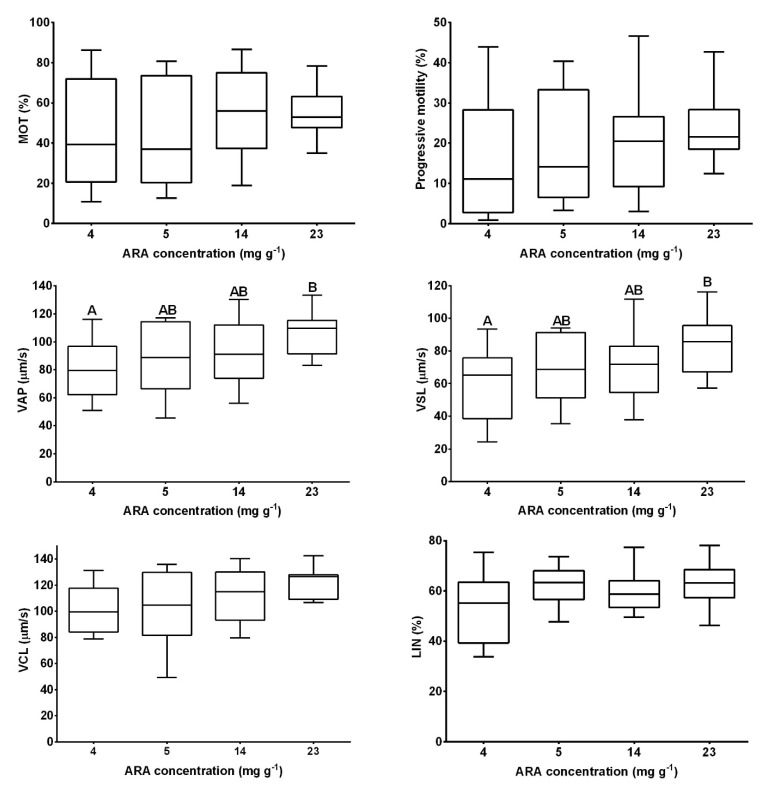
Medaka sperm motility parameters after three weeks of experimental diet feeding with different amounts of ARA as follows: 4, 5, 14, 23 mg ARA g^−1^ of total fatty acid (TFA). Data are expressed as means ± standard deviation (SD) (*n* = 15). Following sperm motility parameters are given: The percentage of motile sperm (MOT, %), progressive motility (%), average path velocity (VAP, μm s^−1^), straight line velocity (VSL, μm s^−1^), curvilinear velocity (VCL, μm s^−1^) and linearity (LIN = 100 × VSL/VCL, %). Different letters above the bars indicate significant differences (*p* < 0.05) between groups.

Initial fertility did not differ significantly between dietary treatments (*p* > 0.05; Figure 1). After the first and second weeks of experimental diet feeding of broodstock, fertility increased in the groups supplemented with 5 and 14 mg ARA g^−1^ TFA and was significantly higher than that of the control group (*p* < 0.05; Figure 1). No differences were noted in the final female fertility (after three weeks of broodstock feeding) (*p* > 0.05; Figure 1; Table 2), while an increase in hepatic COX1 activity was noted with increasing ARA in the diet (*p* < 0.05; Table 2). COX2 activity was not detected in the liver. Egg diameter was similar at the beginning and end of the experiment, ranging from 1.20 to 1.22 mm in groups supplemented with 4 and 14 mg ARA g^−1^ TFA, respectively (*p* > 0.05; Table 2). The fish fed feed supplemented exclusively with ARA (23 mg g^−1^ TFA) exhibited significantly higher sperm motility parameters (VAP, VSL) than the control group fish (*p* < 0.05; Figure 2).

### 3.2. Effect of the ARA Diets on Embryos

**Figure 3:**
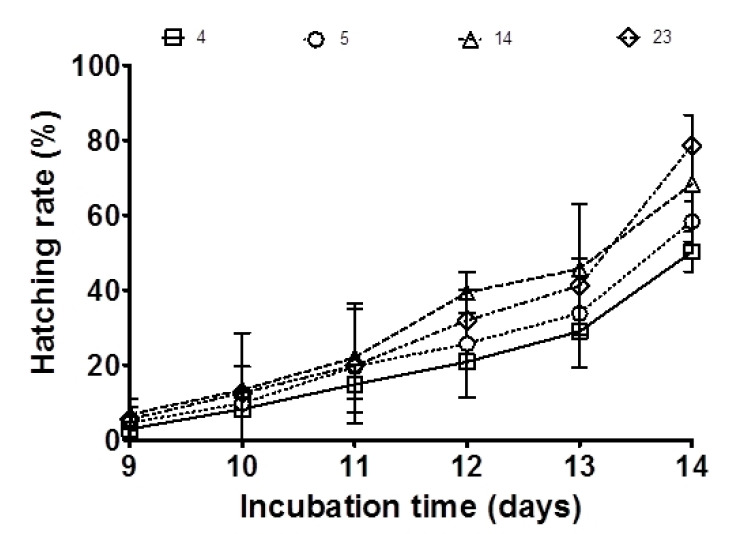
Effect of ARA on the hatching rate derived from medaka broodstock fed diets with different amounts of ARA (4, 5, 14, 23 mg g^−1^ TFA) for three weeks. The values are means ± standard deviation (SD); each dietary treatment was carried out in triplicate (*n* = 3).

Initial embryo survival ranged from 56% ± 17.93 (in the 23 mg ARA g^−1^ TFA group) to 62% ± 18.74 (in the control group). Medaka broodstock fed feed supplemented with the highest amount of ARA for three weeks displayed an increase in embryo survival to 83% (*p* < 0.05; Table 2), while in the control group, embryo survival did not change after three weeks, remaining at 62%. Moreover, larval hatching rates were positively related to ARA content in broodstock feed (range 50.2–78.6%; *p* < 0.05; Figure 3; Table 2). Control group embryos displayed the lowest hatchability rate at 50%. In contrast, the group fed 23 mg ARA g^−1^ TFA revealed a significantly higher hatchability rate (79%).

### 3.3. Effect of the ARA Diet on Larval Quality

**Figure 4:**
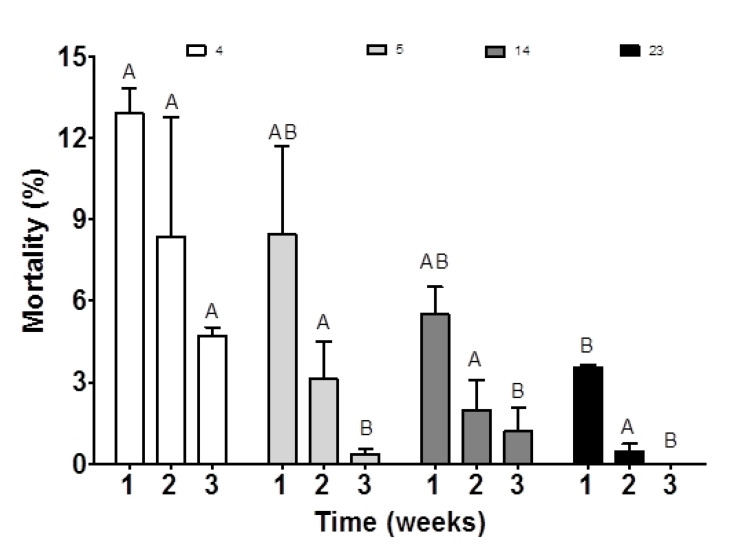
Larval mortality derived from medaka broodstock fed diets containing different amounts of ARA (4, 5, 14, 23 mg g^−1^ TFA) for three weeks. The values are means ± standard deviation (SD) of each dietary treatment in triplicate (*n* = 3). Different letters above the bars indicate significant differences (*p* < 0.05) between groups in the given week of rearing.

Supplementing broodstock feed with a high amount of ARA (23 mg g^−1^ TFA) resulted in significantly increased larval body weight after hatching (*p* < 0.05; Table 2). After three weeks of rearing, the mean body weight of larvae ranged from 2.80 ± 0.78 mg in the control group to 7.60 ± 1.13 mg in the 14 mg ARA g^−1^ TFA group (*p* < 0.05; Table 2). Offspring mortality in the control group at 7, 14 and 21 dph (day post hatch) was higher than that of all other groups (Figure 4) and affected the highest total cumulative mortality for this group significantly (Table 2; *p* < 0.05).

### 3.4. Immunohistochemical Localization of COX2

**Figure 5:**
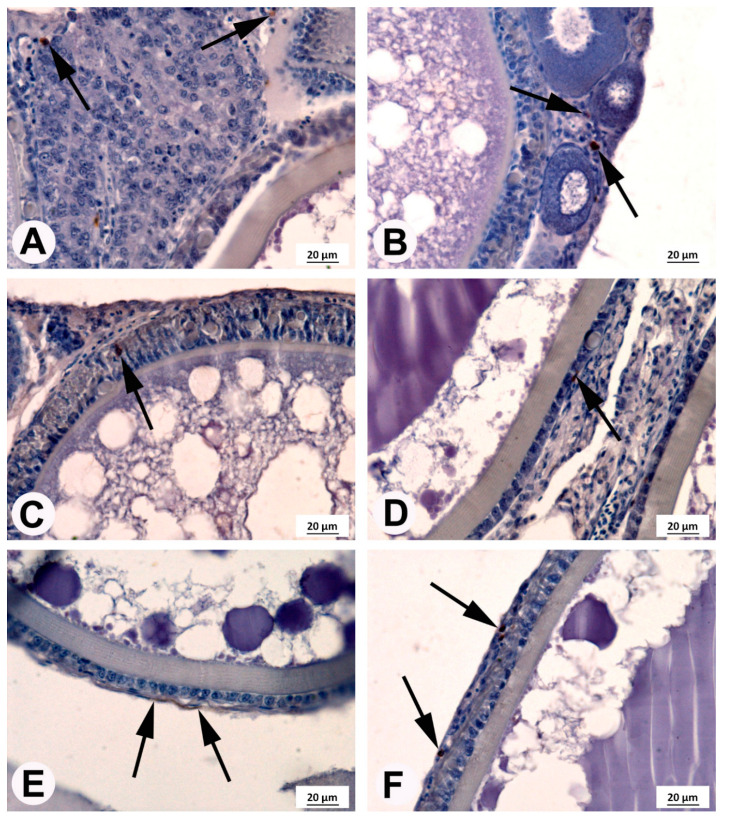
Localization of cyclooxygenase 2 (COX2)-positive cells (arrows) in the medaka ovaries.(**A**) Germinal epithelium; (**B**) growing follicles without the COX2-positive reaction and the COX2-positive cells were localized in germinal epithelium; (**C**) oocyte of medaka from control fish fed 4 mg ARA g^−1^ of TFA at stage VII (vitellogenic phase) with COX2-positive cells present in the follicular layers; (**D**) oocyte of medaka from fish supplemented with 5 mg g^−1^ of TFA at stage IX (postvitellogenic phase) with COX2-positive cells present in the follicular layers; (**E**) oocyte of medaka from fish supplemented with 14 mg g^−1^ of TFA at stages VIII (vitellogenic phase) with COX2-positive reaction in theca cells; (**F**) oocyte of medaka from fish supplemented with 23 mg g^−1^ of TFA at stage IX (postvitellogenic phase) with COX2-positive cells present in follicular layers.

**Figure 6:**
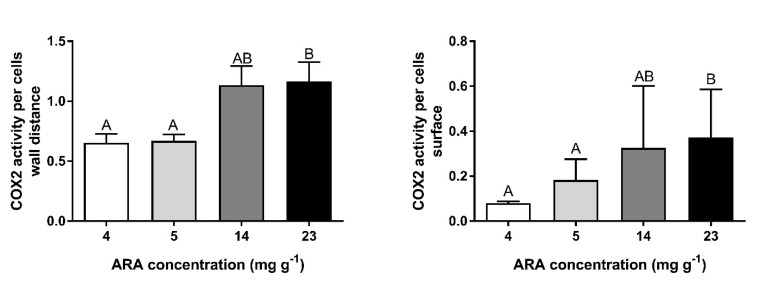
COX2-positive cells in the germinal epithelium and follicular layers in ovary of medaka females fed experimental diets containing 4, 5, 14, 23 mg ARA g^−1^ of TFA. Data are expressed as means ± standard deviation (SD) (*n* = 15). ^A,B^ Different letters above the bars indicate significant differences (*p* < 0.05) between groups.

COX2-positive cells were found in the ovaries of fish from each experimental group (Figure 5). The number and localization of these cells at different stages of maturity of the female germinal cells line varied. In germinal cells and small follicles, such as the primordial follicles and previtellogenic follicles located beneath the germinal epithelium of fish from each experimental group, several COX2-positive cells were detected (Figure 5A). However, in all tested fish, around the growing follicles in the previtellogenic phase, a COX2-positive reaction was observed only in the germinal epithelium (Figure 5B). The large follicles in the vitellogenic phase at stages VII and VIII and in the postvitellogenic phase at stage IX were surrounded by granulosa cells and theca cells. The COX2-positive reaction in granulosa and theca cells was found in all fish tested. The highest COX2-positive cell quantity was observed in females fed feed with ARA of 23 mg g^−1^ TFA (Figure 6).

## 4. Discussion

Our study indicates that supplementation of ARA in the medaka diet to the level of approximately 23 mg g^−1^ of TFA elevates cyclooxygenase activity, sperm motility, hatching rate, embryo growth and survival. Dietary arachidonic acid affects both males and females and, in this way, greatly influences offspring quality.

The addition of ARA to feed changes the fatty acid ratio. In our study, lower values for the ratios EPA/ARA (1.7–3.6 vs. 7.5–8.5) and EPA + DHA/ARA (3.8–6.4 vs. 16.5–19.2) in the 23 mg g^−1^ of TFA ARA diet were followed by improvements in reproduction parameters, higher liver COX1 activity and greater numbers of COX2-positive cells in the ovary. These effects can be related to a high capacity to accumulate ARA, thus decreasing the EPA/ARA and EPA + DHA/ARA ratios in the fish body, as reported by [11]. The amount of these FAs affects ARA conversion to active eicosanoid metabolites, i.e., prostaglandins of series II (PGE2) [20]. Prostaglandin PGE2 was identified as an important PG in the medaka ovary [13], but relative production of PGE2 reflected the relative cyclooxygenase utilization of ARA over EPA or DHA [20]. Our earlier studies have shown egg and larval quality dependence on ARA bioconversion capability and the activity of COX1 [21]. The results of this study indicate a regulatory scheme for COX1 in the liver and COX2 in the ovary by dietary ARA and leads to the conclusion that reproductive parameters are clearly under the control of COX1 and COX2 substrate preference for ARA.

In males, we found that sperm obtained from the group of fish fed with the 23 mg g^−1^ of TFA ARA diet showed higher sperm velocities (VAP and VSL) compared to control group. It was shown previously [22] that inhibition of COX enzymes might influence the sperm velocity and linearity. Therefore, it is plausible that the positive effect of the 23 mg g^−1^ of TFA ARA diet on sperm velocities might be related to the higher level of their metabolites produced by COX-mediated reactions of ARA. Higher availability of ARA might therefore facilitate better sperm quality in medaka. Higher sperm velocity might result in higher fertility as sperm velocity is the parameter affecting fertilization success in fish [23]. Therefore, it seems plausible that feeding medaka broodstock with feed containing a high level of ARA might be beneficial for their reproduction parameters.

In females, dietary supplementation with ARA improves or induces COX1 activity in the liver (this study). ARA is the preferred substrate for COX [24], thus hepatic COX1 activity (this study) and COX2 gene expression [25] are dependent on ARA availability. On the other hand, the activity and/or expression of COX can be inhibited by some environmental factors such as salinity or drugs [21,26,27]. It has been postulated that COX2 activity is important for ovarian function in fish [13]. Therefore, considering our results and those of Petersen et al. [28], who found that ARA treatment led to a sustained expression of COX2, a high amount of ARA in the broodstock diet can be efficiently converted into eicosanoid precursors during the spawning season.

The inhibition of COX1 and COX2 resulted in a decrease in the number of mature oocytes in the medaka ovary [22], due to the effect on ARA bioconversion [14]. It is possible that COX1 activity, which increases with ARA diet supplementation (this study), can initiate the maturation of oocytes, while COX2 expression in the mature ovary, which is essential for successful ovulation [13], has an impact on fertility at the beginning of the reproductive phase (this study). In our experiment, after the first and second week of dietary exposure to supplemental ARA, female fertility increased in groups fed 5, 23 and 14 mg ARA g^−1^ TFA from baseline, while in the control group, female fertility was unchanged in the first week and began to increase by the second week. So, developing oocytes could be initiated at the beginning of spawning by diets supplemented with ARA.

During the reproductive season, the medaka ovary contains oocytes in all stages of oogenesis and only fully grown follicles with large oocytes ovulate [19]. It is possible that ≥5 mg ARA g^−1^ of TFA in the diet increased female fertility at the beginning of spawning due to the presence of COX2 protein in oocytes ([13], this study). We detected the COX2 protein in the ovaries of medaka, however, this localization was nonhomogenous. In the germinal epithelium, few COX2-positive cells were observed, whereas most of the COX2-positive cells were detected in follicular layers, surrounding a large follicle comprising a granulosa cell layer and a theca cell layer. This localization is similar to one described by Hales et al. [29] in the hen (*Gallus domesticus*) ovary and by Richards et al. [30] in the mammalian ovary, confirming that the presence of COX2 protein in the ovary is involved in ovulation [13]. Our study showed an elevation in the area occupied by COX2-positive cells in the follicular layers of ovaries after three weeks of dietary supplementation with ARA. Although final fertility was the same in all dietary treatments (this study), the ovary histology results of the model organism (medaka) suggest that ARA diets in broodstock could adjust COX2 protein expression (indicted by Furne et al. [25]) and, in this way, modulate reproductive physiology in vertebrates [31] and gamete quality in fish (this study).

One of the hypotheses of our study was that the highest embryo survival rate could be related to the expression of various genes in response to the nutritional environment (ARA effects reported in animals by Alves Martins et al. [32]). Dietary ARA supplied at up to 0.1–2% has an impact on COX1 activity and COX2 protein localization in medaka broodstock (this study) and the gene expression of lipase, phospholipase and cyclooxygenase in gilthead seabream (*Sparus auratus*) and sole larvae (*Solea senegalensis*) [32,33]. Lipase, phospholipase and cyclooxygenase are responsible for the synthesis of eicosanoids, including PGE2. PGE2 is currently known to be an essential PG in the medaka ovary [13], and specific PGE2-dependent receptors play essential roles in gastrulation during embryogenesis in zebrafish (*Danio rerio*) [34]. In mammals, an isoform of the COX2 enzyme is responsible for embryo development [35]. Moreover, efficient synthesis of eicosanoids is ARA dose dependent and increases intracellular calcium levels, which facilitates enzymatic conversion in cells [36,37,38]. The consequence may be an effect on embryo development. Therefore, the highest embryo survival and rate of hatching would be found in the 23 mg ARA g^−1^ TFA supplementation group.

In our study, ARA in broodstock diet (14 and 23 mg g^−1^ TFA, which represent 1.4% and 2.3%) had a positive effect on the survival and body mass of offspring, which was evident after three weeks of rearing. Fish growth and survival depend on lipid metabolism, which is related to dietary ARA amount [32]. A larval diet containing 1.7% ARA significantly increased the weight and length of sole larvae compared with diets containing 0.4% ARA [32]. Although a broodstock diet supplemented with ARA (5–23 mg ARA g^−1^ of TFA) also affected the survival rate of medaka offspring, larvae diets enriched with ARA (range 0.4–1.7%) did not change the sole larvae survival rate [32]. The positive influence of broodstock diet on offspring survival compared with direct feeding of offspring was found for the use of other dietary supplements [39]. Although Nath et al. [11] have shown that a high ARA content in the diet (*Artemia* sp.; range 0.3–3.5%) of guppy (*Poecilia reticulata*) fry resulted in lower mortality rates in fish, it should also be noted that too high a level of ARA in the diet can harm the immune response and, consequently, may cause fish mortality [11,40]. The positive effect of ARA was not manifested in the first two weeks of the medaka offspring rearing in the group fed 0.5 and 1.4 mg ARA g^−1^. After 3 weeks of larval rearing, mortality was lower in all experimental groups as compared to the control one. It is possible that lower levels of ARA in the broodstock diet can positively influence the adaptation process of offspring and consequently increase larvae survival at later stages of oogenesis, whereas higher levels could also result in the improvements at the embryo development stages. Further studies are necessary to address this hypothesis.

Our results indicate that ARA enrichment of broodstock diet influences the growth and survival of offspring to a greater extent than feeding the larvae this diet. The absence of a negative effect of ARA as 2.3% of TFA in our experimental diet may indicate the absence of a negative influence on the immune systems of both broodstock and their offspring. It may be possible to improve the immunological parameters in future studies.

In summary, the present study reports for the first time increased activity of COX1 and ovarian localization of COX2 protein associated with a high amount of ARA in the fish diet. These results suggest that ARA bioconversion activity during the reproductive season via COX1 and COX2 activity is involved in female fertility. This study has established that 23 mg ARA g^−1^ TFA in broodstock diets results in the highest rates of an embryo and larval survival. These results are the basis for future investigations to define the mechanisms of the role of ARA added to larval diets in the first stage of oogenesis.

## 5. Conclusions

The results have shown that arachidonic acid in the broodstock diet improves sperm motility parameters. The cyclooxygenase activity in the liver of females increased with the amounts of arachidonic acid in diets. The arachidonic acid bioconversion activity during the reproductive season via cyclooxygenase activity is involved in female fertility and embryo and larval survival and growth.

## Figures and Tables

**Table 1:** Selected fatty acid (FA) composition (mg FA g^−1^ of total FA) of experimental diets used for medaka broodstock feeding.The used abbreviations are as follow: SFA (saturated fatty acids); MUFA (monounsaturated fatty acids); PUFA (poly-unsaturated fatty acids ); LC-PUFA (long chain poly-unsaturated fatty acids); EPA (eicosapentaenoic acid); ARA (arachidonic acid); DHA (docosahexaenoic acid).

Fatty Acids mg FA g^−1^ of total FA	Experimental Diets ^a^			
	4	5	14	23
C14:0	30.45	30.19	29.33	29.28
C16:0	171.16	168.82	165.76	165.14
Σ SFA ^b^	372.21	365.35	363.18	358.86
C16:1	31.68	31.73	30.83	30.70
C18:1 n-9	183.21	178.89	175.79	171.81
Σ MUFA ^c^	275.08	271.80	267.32	264.19
C18:2 n-6	219.24	226.33	219.19	222.62
C18:3 n-3	24.27	24.91	24.19	24.34
C20:4 n-6	4.37	5.16	13.69	22.89
C20:4 n-3	3.65	3.74	3.67	3.75
C20:5 n-3	37.26	38.48	37.91	38.49
C22:5 n-3	8.99	9.00	8.99	9.15
C22:6 n-3	46.52	46.48	49.96	47.45
ΣPUFA ^d^	352.71	362.85	369.50	376.95
Σ n-3 LC-PUFA ^e^	96.42	97.69	100.52	98.84
EPA/ARA	8.53	7.46	3.61	1.68
DHA/ARA	10.64	9.01	3.65	2.07
EPA + DHA/ARA	19.16	16.47	6.42	3.75

^a^ 4—commercial feed (Tetra Min, Germany); 5, 14, 23—commercial feed supplemented with arachidonic acid (ARA ≥99%; Sigma-Aldrich, St. Louis, MO, USA) ^b^ Total saturated FA—14:0, 15:0, 16:0, 18:0, 20:0, 22:0 ^c^ Total monoenes FA—C14:1, C16:1, C17:1, C18:1 *cis*9, C18:1 *cis*11, C20:1 n-9, C21:1 n-7, C22:1 n-11, C22:1 n-9 ^d^ Total polyenes FA—16:2, 16:4, 18:2 n-6, 18:3 n-3, 18:3 n-4, 18:4, 20:2, 20:3 n-6, 20:4 n-6, 20:3 n-3, 20:4 n-3, 20:5 n-3, 21:5, 22:5 n-6, 22:5 n-3, 22:6 n-3 ^e^ Total long chain polyenes n-3 FA—20:4 n-3, 20:4 n-3, 20:5 n-3, 22:5 n-3, 22:6 n-3.

**Table 2:** Reproduction parameters of medaka obtained from broodstock fed experimental diets after three weeks of rearing.

	Dietary Treatments (mg ARA^−^g^−1^ TFA) ^1^			
	4	5	14	23
*Broodstock*				
Body weight (BW, g)	0.45 ± 0.07 ^a^	0.41 ± 0.08 ^a^	0.42 ± 0.07 ^a^	0.43 ± 0.08 ^a^
Body length (TL, cm)	3.35 ± 0.29 ^a^	3.34 ± 0.24 ^a^	3.36 ± 0.19 ^a^	3.40 ± 0.36 ^a^
Fertility (pcs. egg female^−1^)	22.54 ± 5.76 ^a^	21.47 ± 4.10 ^a^	24.70 ± 6.33 ^a^	22.6 ± 4.12 ^a^
Cyclooxygenase activity (COX1, U mg protein^−1^)	10.2 ± 4.2 ^a^	11.8 ± 3.8 ^a^	14.5 ± 5.9 ^b^	13.8 ± 3.6 ^b^
Size of oocyte (mm)	1.20 ± 0.01 ^a^	1.21 ± 0.02 ^a^	1.22 ± 0.01 ^a^	1.21 ± 0.01 ^a^
Embryo survival (%)	62.01 ± 10.97 ^a^	68.36 ± 6.65 ^ab^	65.29 ± 3.24 ^ab^	83.17 ± 5.27 ^b^
Hepatosomatic index (HSI, %)	6.76 ± 1.05 ^a^	7.05 ± 1.16 ^a^	7.27 ± 1.75 ^a^	6.25 ± 1.26 ^a^
*Larvae*				
Hatching rate (%)	50.24 ± 5.36 ^a^	58.32 ± 5.42 ^ab^	75.16 ± 7.23 ^b^	78.58 ± 12.15 ^b^
Initial body weight (BW, mg)	0.40 ± 0.03 ^a^	0.45 ± 0.12 ^ab^	0.45 ± 0.21 ^ab^	0.66 ± 0.10 ^b^
Initial body length (TL, mm)	4.73 ± 0.04 ^a^	4.86 ± 0.09 ^a^	4.88 ± 0.03 ^a^	4.87 ± 0.09 ^a^
Final body weight (BW, mg)	2.80 ± 0.78 ^a^	4.59 ± 0.94 ^ab^	7.60 ± 1.13 ^c^	5.96 ± 0.59 ^bc^
Final body length (TL, mm)	7.98 ± 0.69 ^a^	8.97 ± 0.85 ^a^	8.75 ± 0.61 ^a^	8.49 ± 0.51 ^a^
Total mortality (%)	20.39 ± 5.39 ^b^	9.34 ± 4.78 ^a^	9.47 ± 2.04 ^a^	4.04 ± 1.93 ^a^

^1^ The values are means ± standard deviation (SD); each dietary treatment in triplicate (*n* = 3). ^a,b,c^ Mean value with different letters in the same row were significantly different (*p* < 0.05).
